# Dramatic Therapeutic Response to Dabrafenib Plus Trametinib in BRAF V600E Mutated Papillary Craniopharyngiomas: A Case Report and Literature Review

**DOI:** 10.3389/fmed.2021.652005

**Published:** 2022-01-26

**Authors:** Morena Fasano, Carminia Maria Della Corte, Marianna Caterino, Mario Pirozzi, Raffaele Rauso, Teresa Troiani, Giulia Martini, Stefania Napolitano, Floriana Morgillo, Fortunato Ciardiello

**Affiliations:** ^1^Oncology, Department of Precision Medicine, University of Campania, Naples, Italy; ^2^Oral Surgery, Multidisciplinary Department of Medical-Surgical and Dental Specialties, University of Campania, Naples, Italy

**Keywords:** craniopharyngioma, BRAF, BRAF mutation V600E, targeted, dabrafenib, trametinib, oncogenic driver

## Abstract

**Background:**

Craniopharyngioma is a rare intracranial tumor, with a high morbidity rate due to its common refractiveness to conventional treatments. BRAF V600E mutation has recently been identified as the principal oncogenic molecular driver of papillary craniopharyngiomas (PCP), one of the two main variants of craniopharyngioma.

**Case Presentation:**

A 49-year-old man with recurrent craniopharyngioma, harboring BRAF V600E mutation, has been treated with targeted therapy based on a combination of a BRAF-inhibitor, dabrafenib (150 mg, orally two times daily), and a MEK-inhibitor, trametinib (2 mg, orally two times daily). Before starting treatment, the patient was symptomatic: he lamented confusion, dysphasia, and intense fatigue, that did not allow him to work normally. After just one cycle of treatment, the patient showed an important clinical improvement, reporting a progressive regression of the basal symptoms, hinting at a rapid and dramatic response, which was confirmed at the first radiological assessment. Thus, treatment was continued and at the time of writing, the treatment is still ongoing (total duration of treatment: 14 months) and it is well tolerated, with very good quality of life: the patient has no limitations in daily activities and he has even been able to restart to work.

**Conclusion:**

The use of targeted therapies—as a clinical practice or in clinical trials—represents an important therapeutic alternative and a great evolution for patients' prognosis vs. the standard of care, historically represented by unselected chemotherapies. The discovery of the BRAF V600E mutation in patients with PCP is very rare, resulting in a lack of data on the efficacy of the combination of dabrafenib and trametinib.

## Introduction

Craniopharyngiomas (CPs) are rare primary brain tumors, that are originated from the embryonic remnants of Rathke's pouch (an invagination at the roof of the developing mouth that evolves into the anterior pituitary gland). CPs can be located either in the sella turcica (intra-sellar CPs) or above it (supra-sellar CPs). They show high levels of morbidity, as they arise in the proximity of critical brain structures and can often compress or infiltrate vital neurological areas (e.g., optic nerves, pituitary gland, and hypothalamus). Visual defects, endocrine deficiencies, namely, panhipopituitarism, cognitive deficits, memory loss, headache, morbid obesity, hyperphagia, and personality changes are common complications caused either by tumor growth and the consequences of treatment with surgery, radiation, or both. Careful analysis of tumor extension and symptoms vs. long term and often irreversible adverse effects is important when considering therapeutic strategies (1, 2).

**Figure 1:**
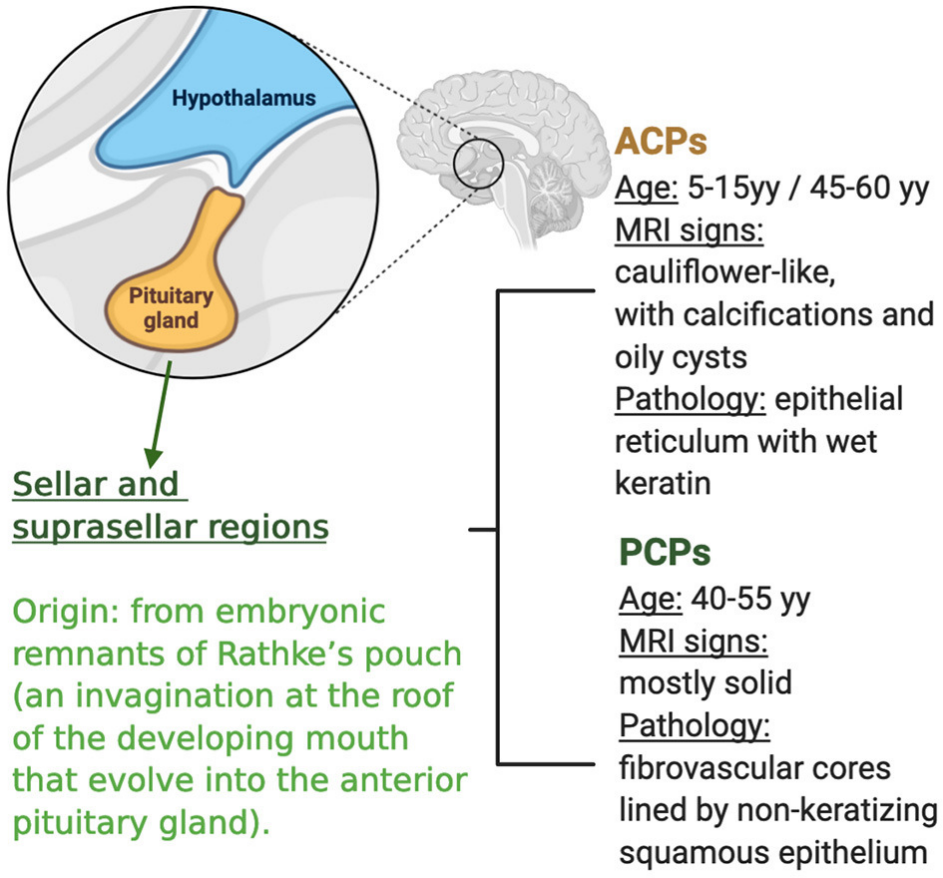
Characteristics of craniopharyngioma and subtypes.

There are two histopathologic variants of craniopharyngioma: adamantinomatus craniopharyngioma (APC) that occurs in both children and adults (with a bimodal age distribution at diagnosis with incidence peaks in the age range of 5–15 years and 40–60 years) and papillary craniopharyngioma (PCP) that occurs almost exclusively in adults, typically between the age of 40 and 55 (1) (Figure 1).

Both histopathologic variants have similar clinical presentation and response to standard treatment, represented by surgery and/or radiotherapy, but there is a high tendency to relapse and worsening of the quality of life. Intracystic chemotherapy (IFNα radiotherapy agents, and bleomicyn) is used in the monocystic subtype of APC.

CPs are characterized by high morbidity due to both hypothalamic and optic chiasm extension, with consequent endocrinological and visual disorders that can usually represent late primary clinical manifestations. CPs presenting as incidental findings are rare (<2% of all CP cases); the diagnosis of childhood CP cases is often late, with a clinical picture characterized by unspecific symptoms connected to increased intracranial pressure, such as nausea and headache (1).

About 1- and 3-year survival rates were found to be better in juvenile patients with small tumors when undergoing subtotal resection and radiotherapy. Conversely, 1- and 3-year survival was worse in the black race (3).

Genomic characterization of APC and PCP showed that each subtype of craniopharyngioma harbors highly recurrent activating gene mutations. In particular, 90% of APCs have mutations in *CTNNB1* consistent with other studies demonstrating that exon three mutations of the beta-catenin gene and WNT pathway activation are important in tumorigenesis of APCs. In addition, over 90% of PCPs have *BRAF* V600E mutations, and the activation of the MAPK pathway is probably the main biological oncogenic driver of these tumors. Moreover, *CTNNB1* and *BRAF* gene alterations are mutually exclusive, being clonal and specific for each subtype. It is not known whether the presence or absence of these molecular alterations correlates with the clinical outcome of patients (4). Unfortunately, *CTNNB1* is not directly targetable with current therapies and often represents a negative prognostic factor for tumor aggressiveness (5), whereas BRAF targeting can be more promising, as suggested by the recent data presented at ASCO 2021 (6).

In view of this, treatment with BRAF-targeted agents may also be an option in PCPs with BRAF mutation. Currently, targeted therapy has been successfully used in treating patients with other tumors with BRAF V600E mutation, namely, melanoma, non small cell lung cancer (NSCLC), papillary thyroid cancer, hairy cell leukemia, colon cancer in combination with Cetuximab. In this study, we present a real-world case report of the efficacy of targeted therapy in PCPs with BRAF mutation.

## Case Description

**Figure 2:**
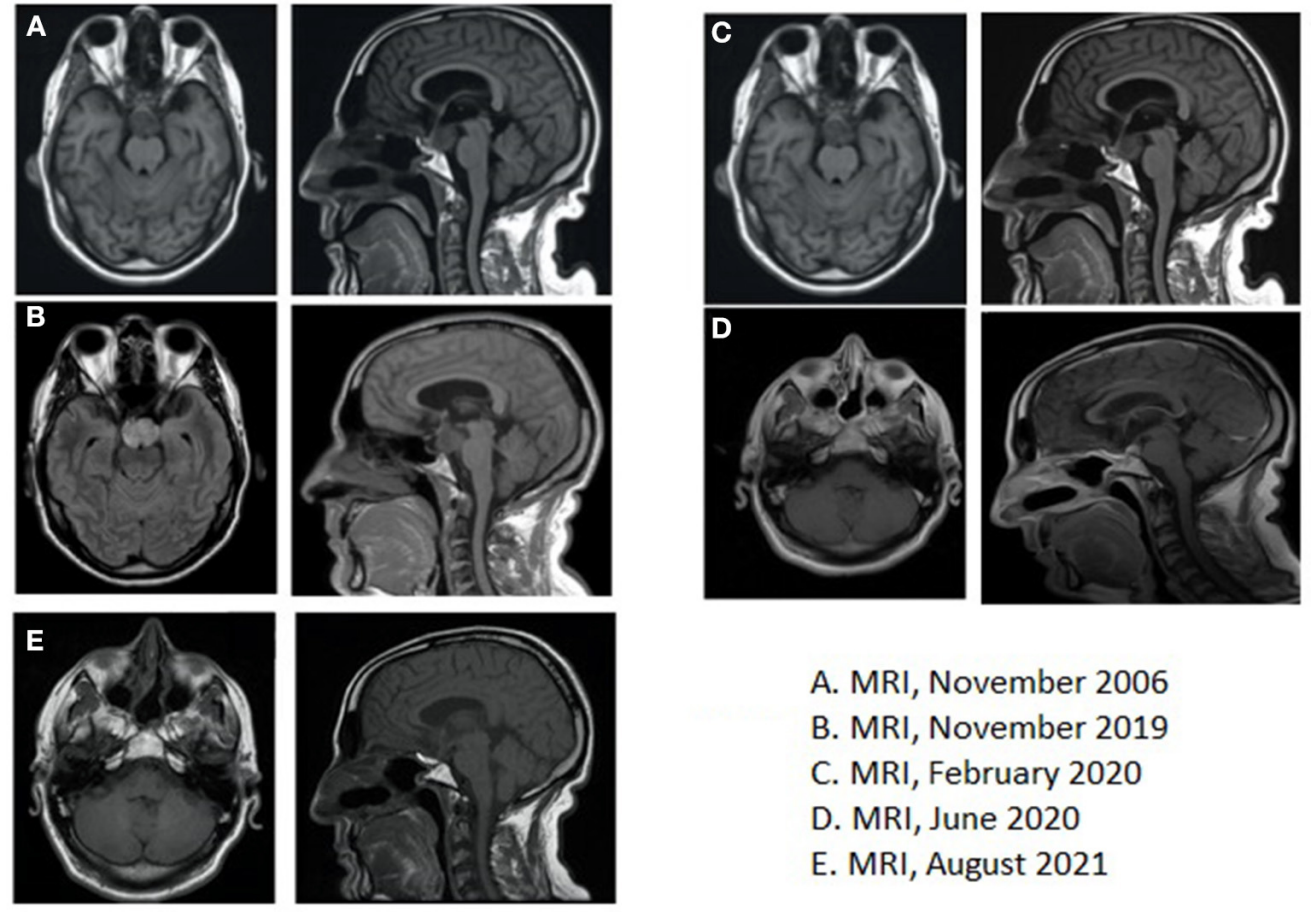
**(A)** Brain magnetic resonance imaging (MRI), November 2006, at the time of the first diagnosis; **(B)** Brain MRI, November 2019, at the time of recurrence; **(C)** Brain MRI, February 2020, showing residual disease after post-second surgery, before starting medical therapy with BRAF- and MEK-inhibitors; **(D)** Brain MRI, June 2020, at the time of partial response after 4 months of medical therapy; **(E)** Brain MRI, August 2021, after 18 months of medical therapy.

In October 2006, a 46-year-old man received brain MRI, advised by a family doctor to investigate the persistency of memory loss and headaches unresponsive to pain killers medications. MRI evidenced a previously unknown infra- and supra-sellar mass of 3.5 × 2.6 cm, with high pressure on the third ventricle and on the interpenducular cistern; with MRI contrast, peripheral highlighting and an 8 mm enhanced solid nodule were found; the imaging was consistent with a diagnosis of cystic craniopharyngioma (Figure 2A).

Subsequently, the patient underwent fronto-temporal trans-cranial excision of the lesion and histological examination confirmed the diagnosis of PCP. In January 2007, the patient was referred by a multidisciplinary committee for discussion of adjuvant radiotherapy (RT), which was confirmed and then administered (total of 50 Gy in 25 fractions). Follow-up visits with clinical and radiological evaluations were periodically performed until 2013, every 3 months when the patient decided to discontinue medical examinations.

In November 2019, for the onset of vertigo and episodes of lipothymia, the patient accessed the emergency department where he performed computed tomography (CT), which showed an iso-dense infra and supra-sellar solid mass of 19 × 23 mm.

Additionally, a brain MRI was also performed, which confirmed the presence of a 25 × 38 × 23 mm lesion that extended to the chiasmatic and inter-peduncular cisterns and the flooring of the third ventricle; mass effect had displaced the pituitary gland to the right and had caused compression of the pons (Figure 2B).

Thus, a second surgery was indicated for avoiding medical complications. Preliminary endocrinological and ophthalmological evaluations were performed and showed no critical signs. Therefore, in January 2020, the patient underwent total resection of the neoformation *via* endonasal endoscopic approach and the histological examination confirmed the recurrence of craniopharyngioma. The postoperative course was complicated by polyuria, mental confusion, dysphasia, ptosis, mydriasis, and ophthalmoplegia, and partially improved by palliative treatment with prednisolone and mannitol. Late surgery-related complications were anosmia and psychomotor impairment. At this stage, considering the young age of the patient and the clinical aggressiveness of the tumor, not controlled by curative treatments with surgery and RT, molecular analysis on the surgical sample was carried out: real-time polymerase chain reaction (PCR) for BRAF gene mutation and then next-generation sequencing analysis for a panel of >300 genes was carried out and the canonical mutation V600E in the exon 15 of BRAF gene was identified as the only relevant mutation (c.t1799a, p. V600E). As previously anticipated, the BRAF mutation had been recognized as a characteristic mutation of PCP, being identified in most, but not all, PCP and never in adamantinomatous craniopharyngioma (ACP). Post-operative brain MRI performed in February 2020 revealed residual tissue with dimensions of 19 × 22 × 19 mm, and after contrast, an inhomogeneous increase in this lesion occurred (Figure 2C). The timeline of the clinical course is highlighted in Table 1.

**Table 1:** Timeline of patient clinical history.

**October 2006**	**Memory loss and headaches**
November 2006	MRI: 35 ×26 mm infra and supra-sellar mass, pressing on third ventricule and interpeduncular cistern
December 2006	Transcranial fronto temporal escision
January → March 2007	Adjuvant radiotherapy 50 Gy
2007 → 2013	FUP (discontinued voluntarily in 2013)
November 2019	Vertigo, unsteady gait, lipothymia CT scan: 19 ×23 mm infra and supra-sellar mass MRI: 25 ×38 ×23 mm, extending to chiasmatic and interpenducolar cisterns and pavement of the third ventricle
January 2020	Endonasal endoscopic exeresis
February 2020	MRI: 19 ×22 ×19 mm residual mass
February 2020	Treatment start: Dabrafenib 300 mg/day + Trametinib 2 mg/day
June 2020	Partial response showed at MRI: 14 ×9 ×5 mm mass (>35% reduction)

Due to the lack of standardized therapeutic approaches, an off-label therapy has been requested, considering BRAF mutation status. In literature, there have been several reports of remarkable responses to BRAF and/or MEK inhibitors (vemurafenib, dabrafenib, and combination therapy) in craniopharyngiomas in various stages, since the discovery that PCPs often harbor BRAF gene mutation (Table 2).

**Table 2:** Published case reports of brain tumors treated with BRAF/MEK-i.

**Report**	**Duration of treatment**	**BRAF-i**	**MEK-i**	**Response**
Brastianos et al. (6)	2 months	Dabrafenib 150 mg bid	Trametinib 2 mg qd for 52 days (starting after 21 days of Dabrafenib)	PR
Aylwin et al. (7)	3 months	Vemurafenib 960 mg	None	Near CR
Roque and Odia (8)	7 months	Dabrafenib 150 mg bid	Trametinib 2 mg qd	CR
Rostami et al. (9)	1$\frac{1}{2}$ months	Dabrafenib 150 mg bid	Trametinib 2 mg qd for 28 days (starting after 21 days of Dabrafenib)	PR
Himes et al. (10)	1 year	Dabrafenib 150–225 mg qd	None	CR

*PR, Partial Response; CR, Complete Response; bid, Bis in Die; qd, Quaque Die*.

The patient started combination treatment with double inhibition of BRAF and MEK with Dabrafenib 300 mg/day and Trametinib 2 mg/day (Table 1).

The patient was consequently evaluated monthly clinically and with a biochemical profile. The treatment was initially well tolerated with no relevant adverse reactions.

At 4 months, laboratory results showed a pathological increase intriglyceride and Cholesterol levels, graded 3 on the CTCAE scale, rarely described during treatment with BRAF/MEK inhibitors. Anti-BRAF and anti-MEK were then suspended temporary and the patient was referred to an endocrinologist: a new medical treatment was established with Fenofibrate and Omega 3 with following the reduction in laboratory levels: oncological treatment was then resumed with dose reduction in accordance to European Medical Agency (EMA) recommendation (Dabrafenib 200 mg/day, Trametinib 1.5 mg/day) when levels reached grade 1, according to CTCAE scale. This was the only adverse event reported by the patient and it did not influence his quality of life. At the time of writing, treatment is ongoing (the total time of treatment until now is 14 months).

First radiological evaluation with MRI after 4 months of treatment showed not only a significant volume reduction of the tumor (14 × 9 × 5 mm vs. 19 × 22 × 19 mm) but also a reduction of the mass effect on the surrounding tissue, for example, realignment of chiasmatic structure (Figure 2D). The last radiological evaluation, performed in August 2021 with MRI after 1 year of treatment, showed overall stability of tumor burden compared to November 2020 (Figure 2E).

## Discussion

Craniopharyngiomas are locally aggressive supra-sellar tumors that often reach a large size before a symptomatic presentation. They compress and infiltrate critical structures, as optic nerves, hypothalamus, and pituitary gland, causing a profound neurological deficit. Standard treatment, namely, surgical resection and radiotherapy may achieve local tumor control, but, unfortunately, poor quality of life often follows these aggressive local treatments due to permanent neurological and endocrine deficits. Tumor control rates after radiotherapy with limited surgery are similar to gross total resection or incomplete resection with radiotherapy, with a >90% overall survival at 10 years. These data do not properly meet with functional results as important brain structures as hypothalamic–pituitary axis, optic nerves, chiasm, and cerebral vascular axis are comprised in the radiotherapy field and can be damaged during the surgery. No standard chemotherapy exists, and when the tumor returns after surgery and radiation, there are no successful therapies to use (1).

Most patients never return to pre-morbid functional levels or good quality of life after multimodality treatment (11). An important part of this morbidity comes from surgery and radiation treatment, which can affect adjacent sensitive visual, endocrine, and neurological structures [i.e., causing permanent primary metabolic alterations, such as obesity (12) and diabetes insipidus (13, 14), and neuro-endocrine disorders, namely, social and emotional alterations (15)]. Therefore, a neoadjuvant or non-operative treatment strategy for cranio-pharingiomas, such as those used for other brain tumors, would be very attractive, even if no data are available until now (9).

Concomitant targeting of BRAF and MEK for PCP treatment, when BRAF V600E mutation is present, is supported by several case reports. In the first published report, single-agent vemurafenib was used in a patient with a PCP with BRAF V600E mutation (7). This report described a tumor that was exceptionally responsive to targeted treatment with vemurafenib, with a near-complete radiological response after 3 months but was short lasting since the tumor relapsed after 6 weeks. The tumor progression seen in the patient treated with single-agent vemurafenib suggested that combining BRAF and MEK inhibition would be preferable for prolonged and durable control of tumor growth (2). In another recently published case report, a man with recurrent PCP with BRAF V600E mutation began dabrafenib therapy and achieved a partial response and clinical benefit after ~12 months of treatment. Therefore, he decided to stop the therapy continuing with follow-up and, 1 year after the interruption of therapy, the patient still shows maintenance of response and clinical benefit (8). Another case report reported a response to anti-BRAF dabrafenib therapy in PCP (10).

While BRAF and MEK targeting is still under scrutiny in PCP, combination therapy or monotherapy is a recognized and standard approach in other several types of solid tumors.

Biologically, the mitogen-activated protein kinase (MAPK) is an essential signaling pathway in several malignancies: alterations in various components of the MAPK pathway, especially in the BRAF gene, have been in fact described in many solid tumors.

BRAF is part of the Rapidly Accelerated Fibrosarcoma (RAF) family (cellular RAF (CRAF), BRAF, and ARAF) of serine/threonine protein kinases and appears as the second tier of the MAP kinase pathway (rat sarcoma (RAS)-RAF- MAPK/ERK kinase (MEK)- extracellular signal-regulated kinases (ERK) cascade), involved in responses to growth signals (16). BRAF itself is the main oncogenic driver in many different types of cancers: mutations have been identified in 45–50% of melanomas, 45–50% of thyroid cancers, 8–10% of colorectal cancers, and 1–5% of NSCLCs.

Targeting MEK is an attractive therapeutic strategy also in non-selected tumors for combination with immunotherapy (17), thus suggesting other future scenarios of evaluation of these drugs also in CP. Furthermore, combining BRAF-inhibition with downstream MEK-inhibition has been shown to be more effective than either therapy alone.

Activating mutations of BRAF lead to consecutive downstream activation of the RAS–MEK–MAPK signaling cascade, promoting cell proliferation, and survival while inhibiting apoptosis, and driving tumor growth (16).

There are fundamentally three classes of BRAF mutation (16):

- Class I mutations are independent of both upstream RAS activation and the need for dimerization (16).
- Class II mutations include several point mutations and fusions that activate MEK through RAS independent dimerization (16).
- Class III mutations are kinase impaired and enhance MAPK signaling through RAS and subsequent CRAF activation (18).

Among these, the V600E mutation, that is, valine substituted by glutamic acid at amino acid 600, a class I mutation, is the most frequent in solid tumors, but other uncommon mutations have been discovered (19, 20).

Following its discovery, several therapeutic approaches targeting BRAF have been studied, first in melanoma (21–25) and then in other cancers. Since resistance is thought to be driven by activation of the downstream MAPK pathway, combination therapy with multiple inhibitions at different sites is the preferred approach to targeted melanoma with BRAF mutation.

In NSCLC, BRAF mutation is rare and associated with the lack of chemo-sensitivity and a worse prognosis in a patient treated with platinum. The European Society of Medical Oncology (ESMO) and the National Comprehensive Cancer Network (NCCN) guidelines recommend the use of BRAF/MEK inhibitors in first or subsequent lines therapy for NSCLC that harbors a V600E mutation, based on phase 2 trials (26).

Similarly, BRAF mutant CRC related respond poorly to standard chemotherapy and show poor prognosis, that may benefit from targeted therapy in combination with Cetuximab, as suggested from recent trials (27, 28).

BRAF mutations, particularly the V600E mutation and KIAA1549-BRAF fusions, are also present in a significant subset of primary brain tumors (29). Positive responses to targeted therapy with RAF inhibitors or with a combination of RAF and MEK inhibitors have been observed predominantly in pediatric brain tumors with BRAF mutation [pylocitic astrocytoma (30), low-grade gliomas (31), and pleomorphic xanthoastrocytoma (32)]. V600E mutation is relatively uncommon in adult glioblastoma (only 3% of cases) and general sensitivity to RAF and/or MEK inhibitors in an adult is not as established as in the pediatric population. Ongoing studies of novel RAF inhibitors are of great importance in neuroncology, due to the fact that not class I MAPK/ERK pathway mutations are relatively common in brain tumors such as low-grade gliomas and pylocitic astrocytoma.

At the last ASCO congress 2021, positive data from a phase II trial of the combination of BRAF and MEK inhibitors (Vemurafenib/cobimetinib) in PCP (Alliance A071601 trial, NCT03224767) have been presented: the objective response was obtained in all but one patient who received at least one cycle of therapy (15/16 patients); median tumor reduction was around 83%. Median PFS was not reached and grade 3 toxicity, which was occurred in 12 patients, was acceptable according to the previous data in other cancer types (6).

These preliminary data further support the potentiality of this type of treatment and the clinical case presented is to be considered as a real-world case for this clinical setting.

Thus, in conclusion, craniophayngioma is considered a tumor with high mortality and frequent recurrence, and its treatments are associated with several complications. Recent evaluations have also shown that craniopharyngiomas have a low frequency of somatic mutations other than BRAF V600E mutation and are not genomically complex. Nevertheless, further experience is needed to define frequency, durability, and extent of BRAF treatment response in PCP (9).

In this present case report, the presence of BRAF mutation was investigated at the time of recurrence, which occurred some years later after the first diagnosis, while the patient was developing significant clinical complications, due to both the locoregional pressure from the relapsing growing mass and after surgical and RT treatments, thus representing a peculiar case of “targeted salvage therapy.”

We believe that the presence of V600E mutation should be investigated as earlier as possible in CP patients, thus giving patients the opportunity to benefit from BRAF and MEK inhibitors treatment. Also, we believe that testing in the neoadjuvant setting of this targeted therapy could be a promising option: shrinkage before definitive treatment with surgery or/and radiation could improve the safety and efficacy of these treatments, potentially reducing the disabling morbidities that often follow current strategies.

We hope that the next year's neo-adjuvant treatment approach may be considered in selected craniopharyngioma patients who underwent biopsy and BRAF mutational status analysis and that results from molecular studies could be available from a greater number of patients to extend knowledge of this rare tumor type.

## Author Contributions

MF and CD wrote the manuscript and supervised the data collection. MC, MP, RR, TT, MF, GM, and SN collected clinical and radiological data and bibliography. FC supervised the full article processing. All authors contributed to the article and approved the submitted version.

## Conflict of Interest

The authors declare that the research was conducted in the absence of any commercial or financial relationships that could be construed as a potential conflict of interest.

## Publisher's Note

All claims expressed in this article are solely those of the authors and do not necessarily represent those of their affiliated organizations, or those of the publisher, the editors and the reviewers. Any product that may be evaluated in this article, or claim that may be made by its manufacturer, is not guaranteed or endorsed by the publisher.
